# Metabolically healthy obesity and physical fitness in military males in the CHIEF study

**DOI:** 10.1038/s41598-021-88728-0

**Published:** 2021-04-27

**Authors:** Sheng-Huei Wang, Pei-Shou Chung, Yen-Po Lin, Kun-Zhe Tsai, Ssu-Chin Lin, Chia-Hao Fan, Yu-Kai Lin, Gen-Min Lin

**Affiliations:** 1 Division of Pulmonary and Critical Care Medicine, Department of Internal Medicine, Tri-Service General Hospital, National Defense Medical Center, Taipei, Taiwan; 2 Institute of Medical Sciences, National Defense Medical Center, Taipei, Taiwan; 3 Department of Internal Medicine, Hualien Armed Forces General Hospital, Hualien County, Taiwan; 4 Department of Critical Care Medicine, Taipei Tzu Chi General Hospital, New Taipei, Taiwan; 5 Department of Neurology, Tri-Service General Hospital, National Defense Medical Center, Taipei, Taiwan; 6 Department of Medicine, Tri-Service General Hospital, National Defense Medical Center, Taipei, Taiwan; 7 Department of Preventive Medicine, Northwestern University Feinberg School of Medicine, Chicago, IL 60611 USA; 8 Department of Medicine, Hualien-Armed Forces General Hospital, No. 630, Jiali Rd. Xincheng Township, Hualien, 971 Taiwan

**Keywords:** Endocrine system and metabolic diseases, Epidemiology, Risk factors

## Abstract

The metabolically healthy obese (MHO) characterized by the absence of metabolic syndrome have shown superior cardiorespiratory fitness (CRF) and similar muscular strength as compared with the metabolically unhealthy obese (MUO). However, this finding might be biased by the baseline sedentary behavior in the general population. This study utilized 3669 physically active military males aged 18–50 years in Taiwan. Obesity and metabolically unhealthy were respectively defined as body mass index ≥ 27.5 kg/m^2^ and presence of at least two major components of the metabolic syndrome, according to the International Diabetes Federation criteria for Asian male adults. Four groups were accordingly classified as the metabolically healthy lean (MHL, n = 2510), metabolically unhealthy lean (MUL, n = 331), MHO (n = 181) and MUO (n = 647). CRF was evaluated by time for a 3-km run, and muscular strengths were separately assessed by numbers of push-up and sit-up within 2 min. Analysis of covariance was utilized to compare the difference in each exercise performance between groups adjusting for age, service specialty, smoking, alcohol intake and physical activity. The metabolic syndrome prevalence in MUL and MUO was 49.8% and 47.6%, respectively. The performance of CRF did not differ between MHO and MUO (892.3 ± 5.4 s and 892.6 ± 3.0 s, *p* = 0.97) which were both inferior to MUL and MHL (875.2 ± 4.0 s and 848.6 ± 1.3 s, all *p* values < 0.05). The performance of muscular strengths evaluated by 2-min push-ups did not differ between MUL and MUO (45.3 ± 0.6 and 45.2 ± 0.4, *p* = 0.78) which were both less than MHO and MHL (48.4 ± 0.8 and 50.6 ± 0.2, all *p* values < 0.05). However, the performance of 2-min sit-ups were only superior in MHL (48.1 ± 0.1) as compared with MUL, MHO and MUO (45.9 ± 0.4, 46.7 ± 0.5 and 46.1 ± 0.3, respectively, all *p* values < 0.05). Our findings suggested that in a physically active male cohort, the MHO might have greater muscle strengths, but have similar CRF level compared with the MUO.

## Introduction

The global prevalence of obesity has increased dramatically over the past few decades, which results in reduced individual life expectancy of more than 10 years and causes enormously social health burden^1–3^. Many guidelines for the diagnosis of obesity was according to body mass index (BMI), but BMI could not precisely estimate the percentage of body fat, location of fat accumulation, and the risk of future obesity associated comorbidities^4^. Early to 1950s, Jean Vague observed that the obese with different body fat distribution may have different propensity for development of atherosclerosis or diabetes mellitus^5^. Therefore, the concept of metabolically healthy obesity (MHO) and unhealthy obesity (MUO) has been established according to numerous observational and interventional studies after decades^1,6,7^.

MUO is one obesity phenotype that 80–90% of obese individuals belong to this category, and MHO is another phenotype that the prevalence is 10–20% in obese individuals and higher in female gender and decreases with aging^6,8^. The body fat disposition in MHO individuals accumulates mainly in the legs and subcutaneous tissues, while that of MUO individuals locates in ectopic regions including visceral tissues and the liver, resulting in abdominal obesity. These two obesity phenotypes are bidirectionally interchangeable by means of weight loss/gain measures, aging, hormone changes, and so on^9–11^. The pathogenesis of MUO is related to adipose tissue dysfunction that chronic positive energy balance decreases subcutaneous adipose tissue expandability, leading to hepatic steatosis and body fat accumulation in visceral tissues^12,13^. The adipose tissue dysfunction and ectopic fat accumulation cause generation of proinflammatory cytokines, disturbance of circulatory singling molecules, lipotoxicity and insulin resistance, which could transit the obesity phenotype from MHO to MUO^6,14^. Several studies have reported that MUO individuals have higher risk of cardiometabolic diseases than MHO individuals^15,16^.

With regard to a comparison of cardiorespiratory fitness (CRF) between MHO and MUO, disparities exist in prior studies. Two large cross-sectional studies conducted by Jae and Ortega reported that MHO individuals had better CRF than MUO individuals, whereas several small studies revealed no differences between the two obesity phenotypes^17–21^. On the contrary, some prior studies reported no differences in muscular strengths between MHO and MUO^19,22–25^. A recent meta-analysis revealed that MHO individuals had higher CRF levels and similar muscular strengths as compared to MUO individuals, while many studies recruited for analysis contained several potential confounders such as different lifestyles, habits, and frequency of illicit behaviors which could not be well adjusted at baseline^26^. Therefore, we conducted a cross-sectional study to investigate the association of CRF and muscle strengths with metabolically healthy and unhealthy lean and obesity in military personnel who lived in the same closed-system environment, and had similar physical activity in Taiwan.

## Methods

### Study population

The whole data of this study was retrieved from the Cardiorespiratory Fitness and Hospitalization Events in Armed Forces (CHIEF) study in Taiwan^27^. The protocol and design of the CHIEF study have been described in detail in prior studies^28–35^. Briefly, this study included 4080 military individuals, aged 18–50 years, who received the annual health examinations including a questionnaire survey for their habitual habits of tobacco smoking (current vs. former/never), alcohol intake (current vs. former/never) and physical activity evaluated by weekly exercise times (each time longer than 30 min) in the past half year (never or occasionally, 1–2 times and ≥ 3 times) in the Hualien-Armed Forces General Hospital, and performed at least one of the annual three exercise tests including 2-min push-ups, 2-min sit-ups and 3 km run test at the Military Physical Training and Testing Center in 2014. As the sample of MHO in females defined as waist circumference < 80 cm and body mass index (BMI) ≥ 27.5 kg/m^2^ was merely 8 cases, all female subjects (n = 411) were excluded for a small sample size which had insufficient power to be analyzed, and thus the male subjects (n = 3669) were left for the following analyses. The present study was approved by the Institutional Review Board of the Mennonite Christian Hospital (No. 16-05-008) in Taiwan and the written informed consents were obtained from all subjects. All methods were performed in accordance with the relevant guidelines and regulations.

### Measurements

The body height and weight of every individual were measured in standing position, and the waist circumference was measured at the midline between the top of the iliac crest and the lowest palpable rib. The definition of BMI was body weight (kg) divided by square of body height (m^2^). Resting blood pressure was assessed over the right upper arm of each participant by an automated blood pressure monitor (FT-201, Parama-Tech Co. Ltd., Fukuoka, Japan). Over a 12-h fasting, venous blood specimens were drawn from each individual to measure concentrations of fasting glucose, triglycerides, and cholesterols on an auto analyzer (AU640, Olympus, Kobe, Japan).

### Metabolic and obese status classifications

For Asian male adults, obesity was defined as BMI ≥ 27.5 kg/m^2^ according to the Taiwan’s Department of Health guidelines^36,37^. The diagnosis of metabolic syndrome was made on the basis of the updated clinical criteria of International Diabetes Federation for the Asian male adults^38^ as the existence of three or more of the following features: (1) abdominal obesity: waist circumference ≥ 90 cm; (2) low fasting high-density lipoprotein cholesterol < 40 mg/dL; (3) high fasting serum triglycerides ≥ 150 mg/dL or on lipid-lowering therapy; (4) high fasting plasma glucose ≥ 100 mg/dL or on antidiabetic therapy (5) high blood pressure ≥ 130 mmHg for systolic and/or ≥ 85 mmHg for diastolic, or on antihypertensive therapy. The status of metabolic unhealth was defined when two components of the metabolic syndrome were presented^6,7^. Four groups were thus classified into the metabolically healthy lean (MHL, n = 2510), metabolically unhealthy lean (MUL, n = 331), MHO (n = 181) and MUO (n = 647).

### Physical fitness tests

Time for a 3-km run test of each participant was used for an evaluation of the level of CRF. The examinees ran 3-km on a flat playground at the Military Physical Training and Testing Center in Hualien without bearing any burden. This running test was held uniformly outdoor at 04:00 PM, and the coefficient of the heat stroke risk formula had to be lower than 40 (the product of outdoor temperature on the Celsius scale and relative humidity (%) × 0.1). In addition, muscular strengths of each participant were separately evaluated by numbers of push-ups and sit-ups within 2 min. The stopping point (2 min) in brief bursts of push-up and sit-up exercises was determined by the findings from other studies^39,40^. These two anaerobic exercises performed on sponge pad were scored by computerized machines. In the push-up test, the examinees obtained score while his back in a line with head and buttocks returned to the initial set level at resting, detected by infrared sensors within 2 min. But the push-up test was discontinued immediately once the body excepting hands and toes touched down on the pad before the time ran out. In the sit-up test, the examinees’ feet were both fixed by the anchors on sponge pad and their hands attached close to the ears. The examinees obtained score when their upper trunk bended forward and the elbows touched the artificial sensors on both thighs.

### Statistical analysis

For the characteristics of each group, categorical variables were expressed as numbers (percentages) and compared by chi-squared test, and continuous variables were presented as mean ± standard deviation (SD) and compared by analysis of variance (ANOVA). Pearson’s correlation coefficients were used to plot the correlation of BMI and waist circumference with each exercise performance. The difference in each exercise performance between groups was estimated with analysis of covariance (ANCOVA), and the results were presented as mean ± standard error (SE). Multiple linear regression analyses were used to determine the relationship of the four groups with each exercise performance. Furthermore, we used multiple logistic regressions to determine the odds ratio (OR) of the best 10% performers and the worst 10% performers in each exercise for comparisons between groups. In model 1, age and service specialty were adjusted. In model 2, current tobacco smoking, current alcohol intake and physical activity were adjusted in addition to the covariates in model 1. A 2- tailed value of *p* < 0.05 was considered significant. SPSS statistical software was used for the statistical analyses (IBM Corp. Released 2013. IBM SPSS statistics for windows, version 22.0. Armonk, NY: IBM Corp.).

## Results

### Baseline group characteristics

Table 1 reveals the characteristics of the four groups. The mean age in MHL was relatively younger than the other groups. Levels of blood pressure were higher in individuals with obesity, BMI ≥ 27.5 kg/m^2^ (MUO and MHO > MUL and MHL) and individuals with abdominal obesity (MUO > MHO and MUL > MHL) than their counterparts. The prevalence of metabolic syndrome in MUL and MUO was 49.8% and 47.6%, respectively (data not shown). There were no differences in the prevalence of physical fitness frequency and current tobacco smoking except that a higher prevalence of active alcohol consumption was observed in MUL.

**Table 1:** Baseline characteristics of the study population (n = 3669).

Characteristics	MHL (n = 2510)	MUL (n = 331)	MHO (n = 181)	MUO (n = 647)	*p* value
Age	28.6 ± 5.9	30.6 ± 5.3	29.7 ± 5.6	31.4 ± 5.4	< 0.01
**Specialty**					
Army	1319 [52.5]	153 [46.2]	82 [45.3]	300 [46.4]	< 0.01
Navy	479 [19.1]	88 [26.6]	43 [23.8]	178 [27.5]	
Air force	712 [28.4]	90 [27.2]	56 [30.9]	169 [26.1]	
Body mass index, (kg/m^2^)	23.3 ± 2.2	25.8 ± 1.4	28.5 ± 0.9	29.2 ± 1.2	< 0.01
(Minimum–maximum)	(15.9–27.4)	(20.3–27.4)	(27.5–32.9)	(27.5–34.8)	
Waist circumference (cm)	79.5 ± 5.8	89.6 ± 4.3	86.5 ± 2.5	94.2 ± 4.0	< 0.01
(Minimum–maximum)	(52.0–89.5)	(75.0–99.0)	(74.0–89.0)	(59.0–117.0)	
Waist to height ratio, WHtR	0.46 ± 0.05	0.51 ± 0.03	0.51 ± 0.05	0.54 ± 0.04	< 0.01
Systolic blood pressure, (mmHg)	116.0 ± 12.4	122.6 ± 12.0	120.6 ± 13.3	124.5 ± 13.2	< 0.01
Diastolic blood pressure, (mmHg)	69.4 ± 9.7	71.5 ± 10.0	73.0 ± 10.6	74.2 ± 10.6	< 0.01
**Blood test**					
Total cholesterol (mg/dL)	169.1 ± 31.9	184.0 ± 33.9	180.5 ± 32.5	188.1 ± 36.9	< 0.01
Serum triglyceride (mg/dL)	94.5 ± 58.6	172.3 ± 181.1	129.9 ± 89.6	161.0 ± 138.7	< 0.01
Fasting plasma glucose (mg/dL)	92.3 ± 10.6	97.4 ± 21.5	93.9 ± 12.0	96.5 ± 16.9	< 0.01
HDL-C (mg/dL)	49.6 ± 9.6	43.4 ± 9.4	45.8 ± 7.5	43.6 ± 8.7	< 0.01
LDL-C (mg/dL)	101.7 ± 28.1	112.1 ± 28.2	112.2 ± 29.9	117.5 ± 31.9	< 0.01
Current alcohol intake	1062 [42.3]	171 [51.7]	80 [44.2]	303 [46.8]	< 0.01
Current smoking	935 [37.8]	122 [37.5]	70 [39.5]	249 [38.8]	0.93
**Physical activity**					
Never or occasionally	536 [21.4]	67 [20.2]	39 [21.5]	127 [19.6]	0.76
1–2 times per week	933 [37.2]	135 [40.8]	62 [34.3]	248 [38.3]	
≥ 3 times per week	1041 [41.4]	129 [39.0]	80 [44.2]	272 [42.0]	

Continuous variables are expressed as mean ± standard deviation, and categorical variables as n [%]. HDL-C, high-density lipoprotein cholesterol; LDL-C, low-density lipoprotein cholesterol. MHL, metabolically healthy lean, defined as body mass index < 27.5 kg/m^2^ and absence of two major components of the metabolic syndrome, according to the International Diabetes Federation criteria for Asian male adults; MHO, metabolically healthy obesity defined as body mass index ≥ 27.5 kg/m^2^ and absence of two major components of the metabolic syndrome; MUL, metabolically unhealthy lean defined as body mass index < 27.5 kg/m^2^ and presence of at least two major components of the metabolic syndrome; MUO, metabolically unhealthy obesity defined as body mass index ≥ 27.5 kg/m^2^ and presence of at least two major components of the metabolic syndrome.

### Pearson’s correlation coefficients

**Figure 1:**
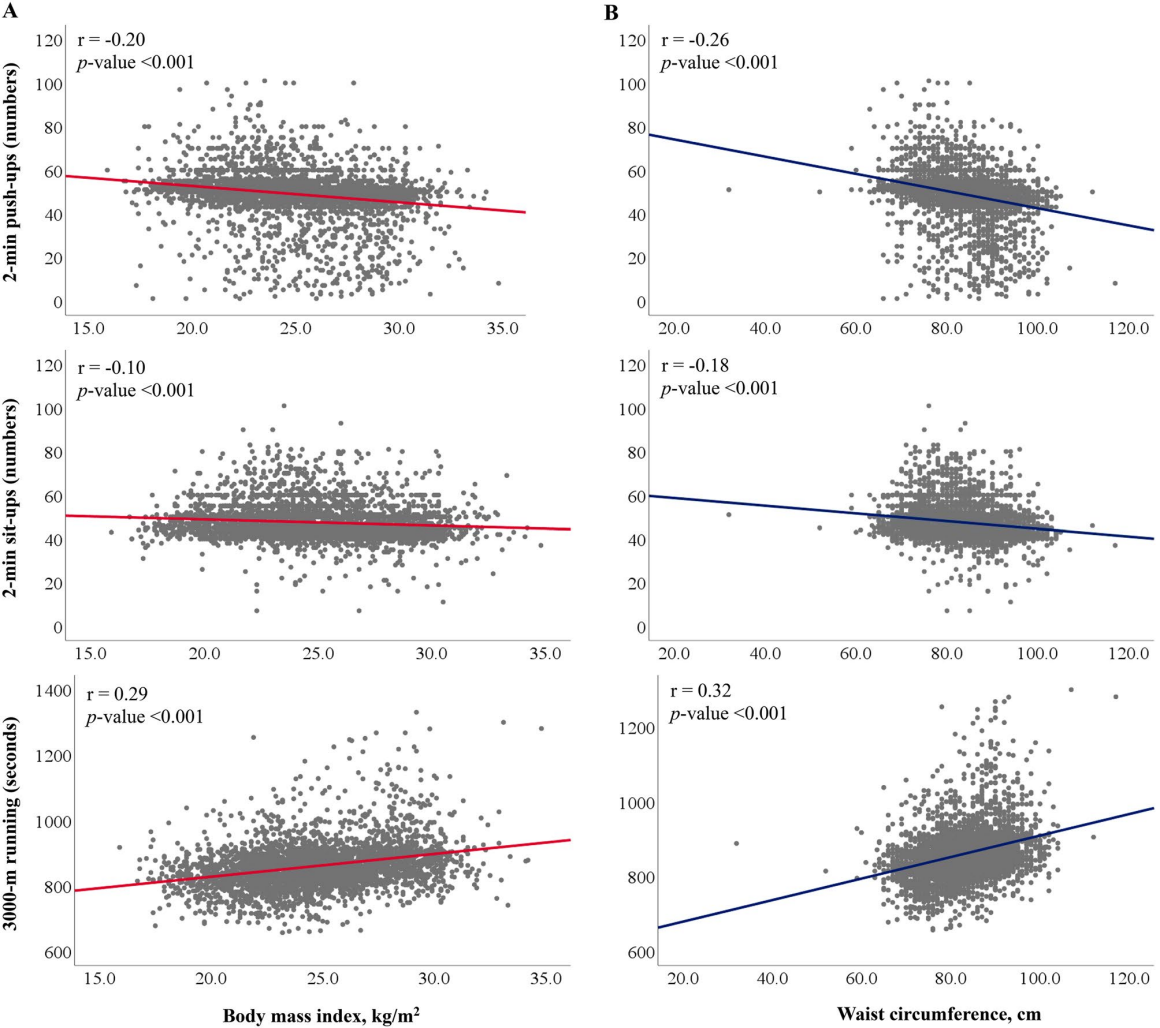
Reveals the Pearson’s correlation coefficients of time for a 3-km run, repetitive numbers of 2-min push-ups and 2-min sit-ups with body mass index and waist circumference, respectively.

Figure 1 displays the Pearson’s correlation coefficients of waist circumference and BMI with the performance of each exercise. Both waist circumference and BMI were positively correlated with time for a 3-km run (r = 0.32 and 0.29, respectively) and inversely with numbers of 2-min push-ups (r = − 0.26 and − 0.20, respectively) and 2-min sit-ups (r = − 0.18 and − 0.10, respectively). All the associations were statistically significant (*p* < 0.001).

### Group mean comparisons

Table 2 shows that the performance of CRF assessed by time for a 3-km run test did not differ between MHO and MUO (892.3 ± 5.4 s and 892.6 ± 3.0 s, *p* = 0.97) which were both inferior to MUL and MHL (875.2 ± 4.0 s and 848.6 ± 1.3 s, all *p* values < 0.05). The performance of muscular strengths evaluated by 2-min push-ups did not differ between MUL and MUO (45.3 ± 0.6 and 45.2 ± 0.4, *p* = 0.78) which were both lower than MHO and MHL (48.4 ± 0.8 and 50.6 ± 0.2, all *p* values < 0.05). On the contrary, the performance of 2-min sit-ups were merely superior in MHL (48.1 ± 0.1) while compared with MUL, MHO and MUO (45.9 ± 0.4, 46.7 ± 0.5 and 46.1 ± 0.3, respectively, all *p* values < 0.05).

**Table 2:** Differences in each exercise performance between various metabolic and obese groups.

	2-min push-ups (numbers)			2-min sit-ups (numbers)			3000-m running (seconds)		
	n	Mean ± SE	*p* value	n	Mean ± SE	*p* value	n	Mean ± SE	*p* value
**Model 1**									
MHL	2495	50.5 ± 0.2	< 0.01^1^	2500	48.0 ± 0.1	< 0.01^1^	2346	849.0 ± 1.4	< 0.01^1^
MUL	326	45.4 ± 0.6	< 0.01^2^	328	46.0 ± 0.4	< 0.01^2^	282	874.5 ± 4.0	< 0.01^2^
MHO	180	48.2 ± 0.8	< 0.01^3^	180	46.6 ± 0.5	0.02^3^	155	892.8 ± 5.4	< 0.01^3^
MUO	640	45.1 ± 0.4	< 0.01^4^	643	46.1 ± 0.3	< 0.01^4^	513	891.7 ± 3.0	< 0.01^4^
			0.01^5^			0.41^5^			0.03^5^
			0.71^6^			0.64^6^			< 0.01^6^
			< 0.01^7^			0.49^7^			0.97^7^
**Model 2**									
MHL	2457	50.6 ± 0.2	< 0.01^1^	2436	48.1 ± 0.1	< 0.01^1^	2311	848.6 ± 1.3	< 0.01^1^
MUL	320	45.3 ± 0.6	< 0.01^2^	322	45.9 ± 0.4	< 0.01^2^	277	875.2 ± 4.0	< 0.01^2^
MHO	176	48.4 ± 0.8	0.01^3^	176	46.7 ± 0.5	0.02^3^	152	892.3 ± 5.4	< 0.01^3^
MUO	634	45.2 ± 0.4	< 0.01^4^	637	46.1 ± 0.3	< 0.01^4^	507	892.6 ± 3.0	< 0.01^4^
			< 0.01^5^			0.33^5^			0.05^5^
			0.78^6^			0.63^6^			< 0.01^6^
			< 0.01^7^			0.41^7^			0.89^7^

^1^Overall *p*-value; ^2^MUL versus MHL; ^3^MHO versus MHL; ^4^MHO versus MHL; ^5^MHO versus MUL; ^6^MUO versus MUL; ^7^MHO versus MUO.

Mean ± SE (standard error) for each exercise performance estimated using analysis of covariance with adjustments for Model 1: age and specialty and Model 2: the covariates in Model 1, physical activity, current alcohol drinking and current tobacco smoking. MHL, metabolically healthy lean, defined as body mass index < 27.5 kg/m^2^ and absence of two major components of the metabolic syndrome, according to the International Diabetes Federation criteria for Asian male adults; MHO, metabolically healthy obesity defined as body mass index ≥ 27.5 kg/m^2^ and absence of two major components of the metabolic syndrome; MUL, metabolically unhealthy lean defined as body mass index < 27.5 kg/m^2^ and presence of at least two major components of the metabolic syndrome; MUO, metabolically unhealthy obesity defined as body mass index ≥ 27.5 kg/m^2^ and presence of at least two major components of the metabolic syndrome.

### Multiple linear regression

Table 3 shows the results of multiple linear regressions of the performance of each exercise between groups. In general, the results were consistent with that presented in Table 2. The CRF level as evaluated by time for a 3-km run was the best in MHL, subsequently followed by MUL, MHO and MUO. With regard to muscular strengths assessed by 2-min push-ups, MHL remained the best and the following were changed to MHO, MUO and MUL. However, for muscular strengths assessed by 2-min sit-ups, there were no differences between MHO, MUO and MUH, except that MHL was better than the others.

**Table 3:** Liner regressions of MUL, MHO and MUO with each exercise performance.

	MUL				MHO				MUO			
	*β* value	95% CI	*p* value	R^2^, %	*β* value	95% CI	*p* value	R^2^, %	*β* value	95% CI	*p* value	R^2^, %
**Model 1**												
*MHL*												
2-min push-ups	− 5.15	− 6.47 to − 3.84	< 0.01	3.8	− 1.06	− 1.91 to − 0.20	0.01	1.5	− 1.78	− 2.12 to − 1.45	< 0.01	5.4
2-min sit-ups	− 2.09	− 3.03 to − 1.15	< 0.01	6.9	− 0.69	− 1.31 to − 0.06	0.03	5.4	− 0.64	− 0.88 to − 0.41	< 0.01	7.4
3000-m running	25.88	17.92–33.84	< 0.01	8.4	21.68	16.41–26.96	< 0.01	8.2	14.13	11.97–16.30	< 0.01	12.9
*MUL*												
2-min push-ups					3.12	0.85–5.40	< 0.01	2.5	− 0.10	− 0.92 to 0.71	0.79	0.6
2-min sit-ups					0.66	− 0.68 to 2.01	0.33	7.6	0.11	− 0.36 to 0.58	0.65	7.7
3000-m running					15.90	0.14–31.66	0.04	2.0	8.24	2.40–14.09	< 0.01	4.9
*MHO*												
2-min push-ups									− 3.38	− 5.43 to − 1.34	< 0.01	2.2
2-min sit-ups									− 0.53	− 1.76 to 0.69	0.39	5.9
3000-m running									0.28	− 15.24 to 15.80	0.97	2.0
**Model 2**												
*MHL*												
2-min push-ups	− 5.16	− 6.47 to − 3.86	< 0.01	6.1	− 1.06	− 1.91 to − 0.22	0.01	3.6	− 1.77	− 2.10 to − 1.44	< 0.01	7.1
2-min sit-ups	− 2.10	− 3.02 to − 1.18	< 0.01	10.3	− 0.70	− 1.31 to − 0.09	0.02	8.9	− 0.64	− 0.87 to − 0.40	< 0.01	10.3
3000-m running	26.21	18.42–34.00	< 0.01	12.5	21.75	16.59–26.92	< 0.01	12.2	14.47	12.35–16.60	< 0.01	16.2
*MUL*												
2-min push-ups					3.00	0.76–5.24	< 0.01	7.1	− 0.11	− 0.92 to 0.70	0.78	2.4
2-min sit-ups					0.65	− 0.67 to 1.98	0.33	11.6	0.11	− 0.35 to 0.58	0.63	9.6
3000-m running					15.07	− 0.34 to 30.49	0.05	7.4	8.56	2.79–14.34	< 0.01	8.0
*MHO*												
2-min push-ups									− 3.34	− 5.38 to − 1.31	< 0.01	3.1
2-min sit-ups									− 0.50	− 1.73 to 0.71	0.41	8.2
3000-m running									0.99	− 14.35 to 16.34	0.89	4.8

Data are presented as *β* and 95% CI (confidence intervals) using Pearson’s correlation coefficients for Model 1: age and service specialty adjustments; Model 2: the covariates in Model 1, physical activity, current alcohol drinking and current smoking adjustments. MHL, metabolically healthy lean, defined as body mass index < 27.5 kg/m^2^ and absence of two major components of the metabolic syndrome, according to the International Diabetes Federation criteria for Asian male adults; MHO, metabolically healthy obesity defined as body mass index ≥ 27.5 kg/m^2^ and absence of two major components of the metabolic syndrome; MUL, metabolically unhealthy lean defined as body mass index < 27.5 kg/m^2^ and presence of at least two major components of the metabolic syndrome; MUO, metabolically unhealthy obesity defined as body mass index ≥ 27.5 kg/m^2^ and presence of at least two major components of the metabolic syndrome.

### Multiple logistic regression

Table 4 reveals comparisons of the possibility as the best 10% and worst 10% performers in each exercise test between groups. With regard to the 2-min push-ups test, the MUO had significantly lower possibility to be the best 10% performer than the MHO group in model 1 and model 2 (odds ratios (OR) 95% confidence intervals: 0.34 (0.19–0.62) and 0.35 (0.19–0.64), respectively). Similarly, the MHO group had a significantly higher possibility than the MUL group to be the best 10% performer in the 2-min push-ups test in model 1 and model 2 (OR: 2.06 (1.08–3.94) and 2.00 (1.03–3.88), respectively) whereas the MHO group had a significantly higher possibility than the MUL group to be the worst 10% performer in 3000-m run test in model 1 and model 2 (OR: 2.15 (1.28–3.62) and 2.14 (1.26–3.64), respectively). For each exercise test, the MHL group had the highest possibility as the best 10% performers and had the least possibility as the worst 10% performers as compared with the other three groups.

**Table 4:** Associations of various metabolic and obese groups with the best 10% and the worst 10% of each exercise performance.

	MUL			MHO			MUO			MHL	MUL	MHO
	OR	95% CI	*p* value	OR	95% CI	*p* value	OR	95% CI	*p* value	Ref	Ref	Ref
**Top 10% of performance level**												
*Model 1*												
2-min push-ups ≥ 60 numbers	0.42	0.26–0.67	< 0.01	0.95	0.55–1.63	0.85	0.31	0.21–0.46	< 0.01	1.000		
2-min sit-ups ≥ 59 numbers	0.61	0.38–0.96	0.03	0.47	0.24–0.91	0.02	0.56	0.39–0.80	< 0.01	1.000		
3000-m running ≤ 783 s	1.07	0.79–1.46	0.63	1.46	0.93–2.31	0.09	1.50	1.21–1.87	< 0.01	1.000		
2-min push-ups ≥ 60 numbers				2.06	1.08–3.94	0.02	0.74	0.41–1.33	0.32		1.000	
2-min sit-ups ≥ 59 numbers				0.96	0.46–2.00	0.92	0.94	0.54–1.63	0.84		1.000	
3000-m running ≤ 783 s				1.16	0.72–1.86	0.52	1.38	0.98–1.94	0.06		1.000	
2-min push-ups ≥ 60 numbers							0.34	0.19–0.62	< 0.01			1.000
2-min sit-ups ≥ 59 numbers							0.95	0.48–1.88	0.90			1.000
3000-m running ≤ 783 s							1.18	0.78–1.80	0.42			1.000
*Model 2*												
2-min push-ups ≥ 60 numbers	0.41	0.25–0.66	< 0.01	0.99	0.57–1.72	0.99	0.31	0.20–0.46	< 0.01	1.000		
2-min sit-ups ≥ 59 numbers	0.59	0.37–0.94	0.02	0.49	0.25–0.96	0.03	0.55	0.39–0.79	< 0.01	1.000		
3000-m running ≤ 783 s	1.07	0.79–1.46	0.62	1.49	0.94–2.37	0.08	1.50	1.21–1.87	< 0.01	1.000		
2-min push-ups ≥ 60 numbers				2.00	1.03–3.88	0.03	0.74	0.41–1.34	0.33		1.000	
2-min sit-ups ≥ 59 numbers				0.96	0.45–2.02	0.92	0.96	0.55–1.67	0.90		1.000	
3000-m running ≤ 783 s				1.18	0.73–1.90	0.48	1.40	0.99–1.98	0.05		1.000	
2-min push-ups ≥ 60 numbers							0.35	0.19–0.64	< 0.01			1.000
2-min sit-ups ≥ 59 numbers							0.98	0.49–1.95	0.96			1.000
3000-m running ≤ 783 s							1.17	0.77–1.79	0.45			1.000
**Bottom 10% of performance level**												
*Model 1*												
2-min push-ups ≤ 37 numbers	3.06	2.21–4.23	< 0.01	1.50	0.85–2.65	0.15	2.99	2.30–3.88	< 0.01	1.000		
2-min sit-ups ≤ 40 numbers	1.88	1.33–2.67	< 0.01	1.70	0.92–3.16	0.08	1.63	1.25–2.14	< 0.01	1.000		
3000-m running ≥ 934 s	1.71	1.15–2.54	< 0.01	2.19	1.27–3.78	< 0.01	2.92	2.23–3.84	< 0.01	1.000		
2-min push-ups ≤ 37 numbers				0.68	0.41–1.15	0.15	0.97	0.69–1.38	0.89		1.000	
2-min sit-ups ≤ 40 numbers				0.72	0.39–1.33	0.30	0.84	0.56–1.25	0.39		1.000	
3000-m running ≥ 934 s				2.15	1.28–3.62	< 0.01	1.68	1.11–2.54	0.01		1.000	
2-min push-ups ≤ 37 numbers							1.41	0.87–2.27	0.15			1.000
2-min sit-ups ≤ 40 numbers							1.18	0.68–2.06	0.54			1.000
3000-m running ≥ 934 s							0.80	0.52–1.23	0.31			1.000
*Model 2*												
2-min push-ups ≤ 37 numbers	3.14	2.26–4.35	< 0.01	1.42	0.81–2.51	0.21	3.01	2.32–3.91	< 0.01	1.000		
2-min sit-ups ≤ 40 numbers	1.85	1.30–2.63	< 0.01	1.56	0.83–2.91	0.15	1.65	1.25–2.17	< 0.01	1.000		
3000-m running ≥ 934 s	1.72	1.16–2.57	< 0.01	2.07	1.19–3.57	< 0.01	2.93	2.23–3.86	< 0.01	1.000		
2-min push-ups ≤ 37 numbers				0.69	0.40–1.17	0.17	0.97	0.68–1.38	0.89		1.000	
2-min sit-ups ≤ 40 numbers				0.74	0.40–1.38	0.34	0.86	0.57–1.29	0.48		1.000	
3000-m running ≥ 934 s				2.14	1.26–3.64	< 0.01	1.66	1.09–2.51	0.01		1.000	
2-min push-ups ≤ 37 numbers							1.39	0.86–2.26	0.17			1.000
2-min sit-ups ≤ 40 numbers							1.19	0.68–2.08	0.52			1.000
3000-m running ≥ 934 s							0.80	0.52–1.24	0.32			1.000

Data are presented as odds ratios (OR) and 95% CI (confidence intervals) using multiple logistic regression analysis for Model 1: age and service specialty adjustments; Model 2: the covariates in Model 1, physical activity, current alcohol drinking and current smoking adjustments. MHL, metabolically healthy lean; MHO, metabolically healthy obesity; MUL, metabolically unhealthy lean; MUO, metabolically unhealthy obesity.

## Discussion

We found some intriguing and important points in this cross-sectional study conducted in a well-controlled military environment. First, it is in accordance expectation that MHL individuals had the best CRF level and muscle strength among the four groups categorized according to BMI and waist circumference. Second, MHO individuals had greater muscle strength assessed by 2-min push-ups than MUO individuals, while the CRF levels of the two groups were similar. Third, MHO individuals had greater muscle strength assessed by 2-min push-ups but less CRF level assessed by time for a 3 km run test than MUL individuals. The last two points were novel findings and have not been reported before.

Several studies reported MHO individuals had higher CRF level compared to MUO individuals, but the recruited individuals living with different lifestyles, habits and environments, which could not be well adjusted^26,41^. However, this study uncovered that MHO and MUO individuals completed 3-km run with similar time. This finding suggested that MUO individuals could achieve the same level of CRF as MHO individuals when performing the same type, frequency and strength of physical activity in the military bases. In addition, it has been well known that MHL individuals have the lowest risk to develop cardiovascular disease compared with other groups partly because of the best CRF level. Whether improving the CRF for MUO individuals by intensifying daily physical activities could decrease the risk of developing cardiometabolic comorbidities needs more evidence to be verified.

Our study demonstrated that MHO individuals might have greater muscular strength but not CRF level than MUL individuals. Some studies have reported that combined aerobic exercise (i.e. long-distance run) and anaerobic exercise (i.e. short-term push-ups) can reduce more abdominal subcutaneous adipose tissue than aerobic or anaerobic exercise alone^42–44^. In addition, the reduction of liver and visceral fat amounts might not differ with regard to the intensity and dose of aerobic exercise which the participants received^45^, and an increase of skeletal muscle mass was observed only in those taking anaerobic (resistant) exercise^45^. It is possible that MHO individuals might frequently receive more combined aerobic and anaerobic exercise training than the MUO and MHL so that MHO individuals had greater muscular strength than their counterparts. By contrast, the greater CRF levels in MUL individuals than MHO and MUO individuals was probably due to their lower BMI levels.

There were some strengths of this study. First, there were sufficient numbers of military males for analyses to detect the differences in the performance of aerobic and anaerobic exercise in the four classified groups. Second, the three kinds of exercise tests were performed in a strict manner, and the process was standardized. Third, all the military males lived in the same environment and received similar training which could minimize the potential confounders to bias the study results. On the other hand, our study existed some limitations. First, this study recruited only male individuals so that the results could not be extrapolated to female individuals. Second, the presence of selection bias could not be excluded, for the participation rate of the military individuals in this study is 66.5%. Finally, for the essence of cross-sectional study, the causality between the status of obesity, metabolic syndrome and exercise performance could not be clarified.

In conclusion, we found that in a physically active cohort, MHO individuals had similar CRF level as MUO individuals, and had better muscle strength than the MUL. Furthermore, we uncovered that MHL individuals had the best CRF and muscle strength levels among all the groups, highlighting the importance of transition to MHL status from metabolically unhealthy or obese status since young adults.

## Abbreviations

CHIEF: The Cardiorespiratory Fitness and Hospitalization Events in Armed Forces study
CRF: Cardiorespiratory fitness
MHL: Metabolically healthy lean
MHO: Metabolically healthy obesity
MUL: Metabolically unhealthy lean
MUO: Metabolically unhealthy obesity
